# Mineralization mechanism of organic carbon in maize rhizosphere soil of soft rock and sand mixed soil under different fertilization modes

**DOI:** 10.3389/fpls.2023.1278122

**Published:** 2023-11-16

**Authors:** Zhen Guo, Jichang Han, Yang Zhang, Huanyuan Wang

**Affiliations:** ^1^ Institute of Land Engineering and Technology, Shaanxi Provincial Land Engineering Construction Group Co., Ltd., Xi’an, China; ^2^ Shaanxi Provincial Land Engineering Construction Group Co., Ltd., Xi’an, China; ^3^ Key Laboratory of Degraded and Unused Land Consolidation Engineering, Ministry of Natural Resources, Xi’an, China

**Keywords:** organic amendments, organic carbon mineralization, fertility characteristics, aggregation structure, bacterial community

## Abstract

**Introduction:**

This article endeavors to investigate the influence of various fertilization methods on the characteristics of rhizosphere soil and organic carbon mineralization in the mixed soil of Mu Us Sandy land under maize cultivation, with the objective of laying the groundwork for low-carbon agriculture and the development of high-quality farmland.

**Methods:**

The research focuses on soft rock and sand composite soil with a 1:2 ratio, and it comprises four treatments: no fertilization (CK), only chemical fertilization (CF), only cattle manure application (MF), and only oil residue application (DF).

**Results:**

The findings revealed that the use of organic fertilizer substantially elevated nutrient content and enzyme activity in the maize rhizosphere soil. Furthermore, it had a notable influence on both soil aggregate diameter and stability. Specifically, the DF treatment led to a significant increase in both soil aggregate diameter and stability. The mineralization rate of organic carbon in the maize rhizosphere soil could be categorized into two distinct phases: a rapid initial decline followed by a slower release. By the end of the incubation period, the cumulative mineralization of organic carbon in the MF, DF, and CF treatments showed a significant increase of 119.87%, 57.57%, and 24.15%, respectively, in comparison to the CK treatment. Additionally, the mineralization rate constants of the DF and MF treatments experienced a substantial rise, with increments of 23.52% and 45.97%, respectively, when contrasted with the CK treatment. The bacterial phyla Actinobacteriota, Proteobacteria, Chloroflexi, Acidobacteriota, and Firmicutes were dominant in the rhizosphere soil bacterial community. Specific genera such as Nocardioides and Sphingomonas showed significant correlations with organic carbon mineralization. The application of different organic fertilizer can improve soil physical, chemical and biological properties, and promote the mineralization process of organic carbon in maize rhizosphere soil.

**Discussion:**

Notably, the DF treatment exhibited the most favorable outcome, improving the overall quality of maize rhizosphere soil while incurring a minimal loss of unit organic carbon. These findings hold significant implications for optimizing field management practices and augmenting soil quality.

## 1 Introduction

Maize (*Zea mays L.*) has traditionally been cultivated in Mu Us Sandy Land. Nevertheless, in recent years, the introduction of land consolidation technology has enabled the expansion of maize cultivation from the southern desert periphery into the inland areas, leading to a transition from scattered to large-scale farming (Liu et al., 2021). Unfortunately, the establishment of extensive maize cultivation areas often involves the flattening of undulating sand dunes and the mixing of sand with other soil types. This process leads to the destruction of native sandy vegetation and disrupts the original spatial and temporal patterns (Gehl et al., 2005). The spatial characteristics of newly reclaimed sandy land do not undergo land grid consolidation, resulting in limited capacity to block and intercept wind-blown sand during the crop growing season, despite an increase in surface vegetation coverage (Zhang et al., 2021a). Temporally, maize, being a single-crop plant, leaves two-thirds of the land surface exposed throughout the year, rendering it highly susceptible to sand erosion and a significant contributor to wind erosion and dust storms (Zhou et al., 2019; Cheng et al., 2021). The unstable nature of the agricultural ecosystem in sandy lands stands in stark contrast to the high-quality agricultural development in the desert oasis ecological area, which has become a prominent topic of discussion and research in academia.

The Mu Us Sandy Land, one of China’s four major sand regions, holds a pivotal role in China’s ecological security framework. Nevertheless, the conventional method of sand consolidation involves the transportation of loess from other regions to the Mu Us Sandy Land, resulting in a significant waste of time, labor, and resources. Moreover, this approach is highly susceptible to environmental degradation (Ywab et al., 2019). In the Mu Us Sandy Land, soft rock and sand coexist relatively independently. The soft rock experiences minimal diagenesis, leading to a state that is dry and hard, resembling stone, but it becomes soft and mud-like when exposed to water. Conversely, sand displays high porosity, weak cementation, low structural strength, and is prone to water and fertilizer leakage (Han et al., 2012; Sun and Han, 2018). Han’s research team has efficiently harnessed the abundant soft rock resources within the Mu Us Sandy Land, blending them with sand to create a composite soil layer that fosters crop growth. By optimizing the sand consolidation technique, they have effectively restored the cultivability of the sandy land while also conserving valuable resources (Wang et al., 2013; Han and Zhang, 2014; Zhang and Han, 2019). According to Zhang et al. (2020), research has established that maintaining a ratio of clay minerals to sand at 1:2 can significantly enhance the texture and organic matter content of sandy soil. This finding holds considerable theoretical and practical importance for sustainable agricultural utilization. Furthermore, according to Sun et al. (2021), a soft rock to sand ratio of 1:2 results in the formation of the largest soil aggregates and the highest maize yields. These findings suggest that this ratio can effectively improve soil structure and yield economic benefits. Nevertheless, even with a composite soil ratio of 1:2, the soil’s fertility level remains low.

In recent years, there has been a growing research focus on the effects of reducing chemical fertilizer usage and increasing the application of organic fertilizer on soil fertility enhancement and changes in organic carbon quality (Mikha and Rice, 2004). Guo et al. (2019a) demonstrated that the long-term application of cow manure organic fertilizer enhances the carbon and nitrogen content within the 0-20 cm soil layer of sandy land. The inclusion of cow dung not only accelerates the decomposition of soil organic carbon but also elevates the content of soil organic carbon and its active carbon pool components, consequently bolstering soil organic carbon sequestration and microbial activity (Qi et al., 2016). Zhou et al. (2019) observed that sandy soil typically exhibits poor fertility, and the introduction of chemical fertilizers to such soil can rapidly augment maize grain yield and water use efficiency. Nevertheless, while a substantial influx of fertilizers can yield short-term increases in maize yield, it also precipitates a series of environmental issues, including aggravated soil degradation and groundwater eutrophication (Ren et al., 2021). Oil residue, as an alternative fertilizer, has the capacity to significantly enhance available phosphorus and organic matter in sandy soil (Kasongo et al., 2011). Oil residue is enriched with carbon, nitrogen nutrients, and trace elements. Following its application, there is a substantial increase in soil organic matter content, leading to a significant improvement in soil fertility as well (Xu et al., 2021). Research has also indicated that oil residue may stimulate the microbial community structure and activity in the soil, thereby promoting maize growth (Abdullah and Shagufta, 2016; He et al., 2021). While it is clear that various fertilization methods have positive effects on increasing soil nutrients, further research is necessary to ensure the sustainability of this nutrient enhancement.

Soil organic carbon mineralization is a crucial biochemical process that plays a significant role in responding to fertilization practices. It is closely linked to material cycling and the release of CO_2_ within soil ecosystems. Recent studies have shown that the complete replacement of traditional fertilizer with 100% organic fertilizer can significantly accelerate the decomposition of organic carbon, thereby reducing the stability of soil organic carbon (Lin et al., 2022). Long-term organic fertilizer application has been demonstrated to improve soil fertility, boost crop yields, and mitigate environmental pollution resulting from the excessive use of chemical fertilizers (Lin et al., 2009). Hence, the selection of the appropriate fertilization method is of paramount importance, considering the specific research objectives and the study’s duration. The rhizosphere, the zone where plants, soil, and microorganisms interact, plays a crucial role in facilitating the uptake of various nutrients, water, beneficial, and harmful substances into the root system, contributing to the material cycling within the biological chain (Rai et al., 2006). To promote sustainable agriculture and ecological restoration of sandy lands, it is imperative to delve into the soil organic carbon mineralization process within the rhizosphere. Furthermore, an exploration of the role of organic carbon mineralization in non- uniform media, such as soils with soft rock and sand complexes, can enhance our comprehension of soil ecosystem stability and functionality. This study aims to: (1) elucidate the impact of various fertilization methods on the nutrient content and organic carbon mineralization within the maize rhizosphere soil, and (2) unravel the physico-chemical-biological mechanisms underlying organic carbon mineralization in maize rhizosphere soil.

## 2 Materials and methods

### 2.1 Basic information of experimental site

The test area was situated at the pilot base in Fuping County, China (109°11′41.63’’E, 34°42′2.47’’N). It was located on the southern edge of the Ordos platform and the slope belt on the northern edge of the Weihe Graben. It falls within the canyon region of the Weibei Loess Plateau and has an elevation ranging from 376 to 439 meters.

**Figure 1:**
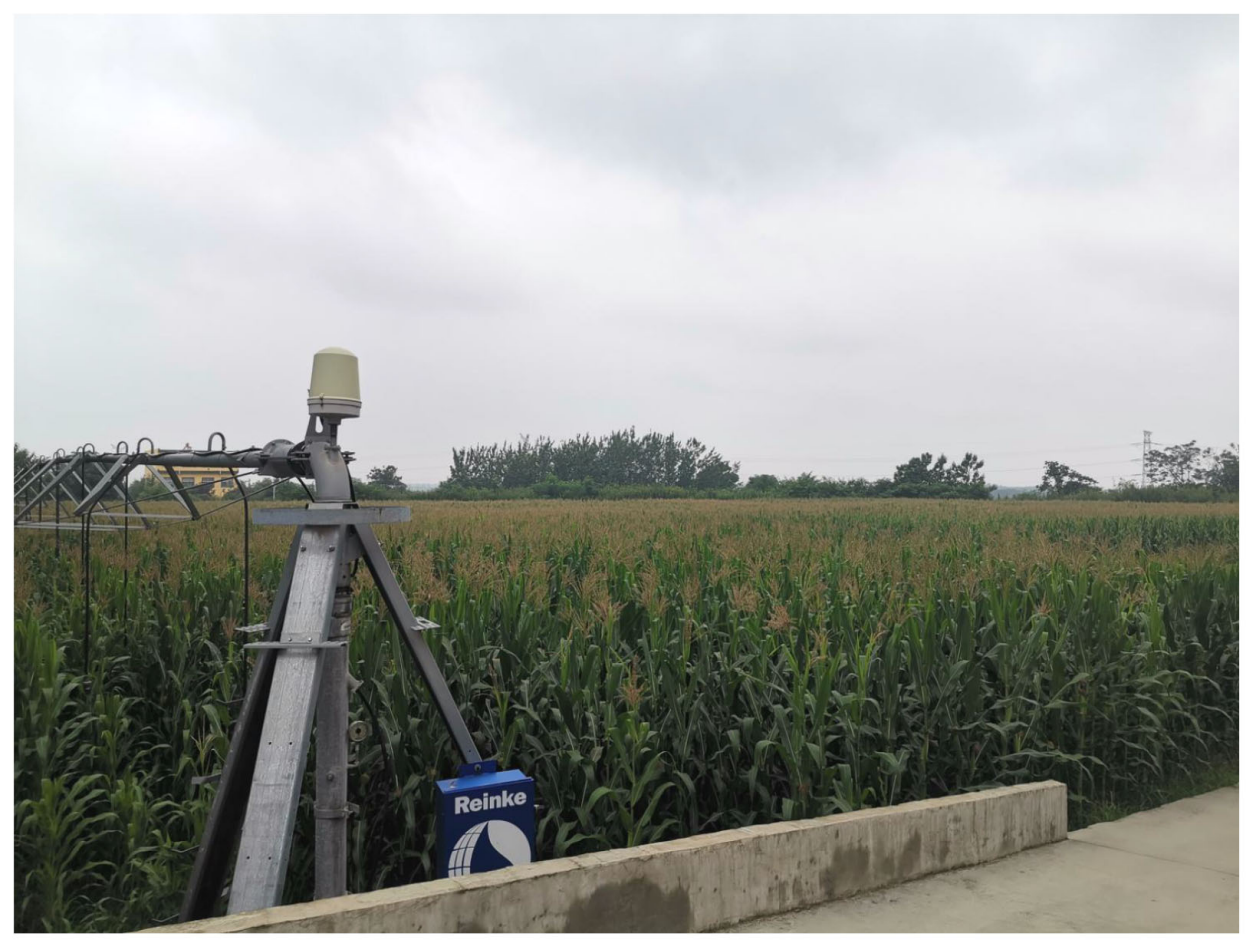
Planting maize in the experimental area and matching with sprinkler irrigation machines.

With an annual sunshine duration of 2472 hours and an annual average sunshine rate of 56%, it was well-suited for cultivating a variety of crops (**Figure 1**). Due to the influence of terrain, altitude, and vegetation, there was a notable temperature variation. Generally, temperatures exhibit an increasing trend in the southeast, decreasing in the northwest, and declining from south to north. The annual average temperature stands at 13.1°C, annual average precipitation was 533.3 mm, and the average annual frost-free period spans 225 days.

### 2.2 Experimental design

**Figure 2:**
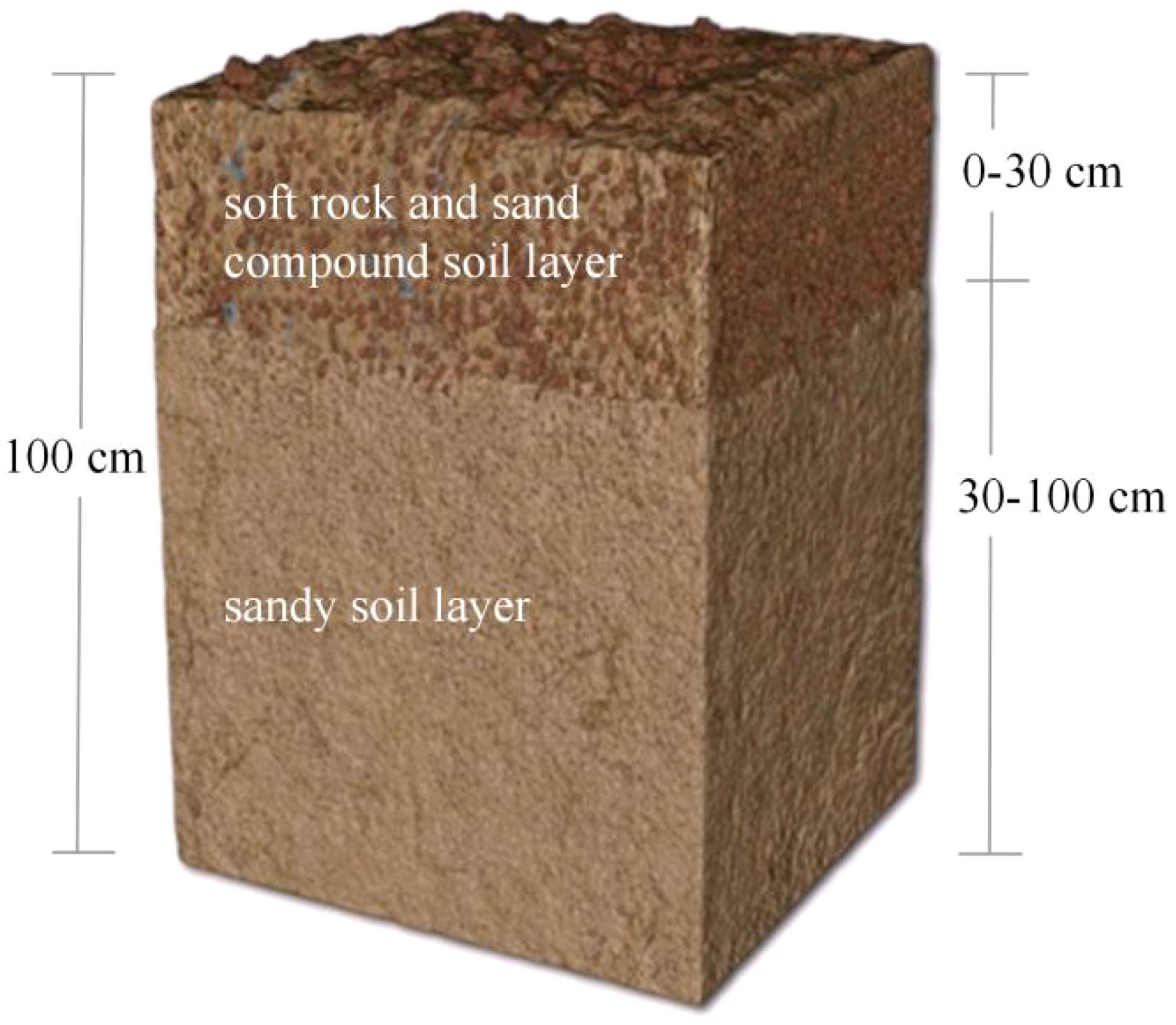
Structural diagram of composite soil model in experimental plot. The soil of 30-100 cm is filled with sandy soil, and the surface layer of 0-30 cm is laid with a mixed layer with a volume ratio of soft rock to sand of 1:2.

To improve the nutrient content of the composite soil, a pilot plot was set up at the fertilizer efficiency research pilot base. The aim was to replicate the land conditions found in the mixed layer of soft rock and sand in the Mu Us sandy land. The test plot was filled with sandy soil to a depth of 30-100 cm. Additionally, the soft rock was mixed thoroughly with sand at a ratio of 1:2 in the surface layer of 0-30 cm (**Figure 2** and **Table 1**). Four fertilization treatments were set up, namely no fertilization (CK), separate application of chemical fertilizer (CF), separate application of cow manure (MF), and separate application of oil residue (DF). The experimental design used was a randomized block design, with three replicates for each trial treatment. The experimental field was planted with artificially seeded maize (Zheng Dan 958). The sowing row spacing of maize was 50 cm, the plant spacing was 30 cm, the seeds were planted in acupoints and the sowing depth was about 5 cm. After sowing, the soil was covered with moisture in time, and one plant was retained in each hole after seedlings were set. All organic materials were applied as base fertilizer in a single application before sowing. The application amount of cow manure (65% organic matter, 1.38% total nitrogen, 0.41% total phosphorus, 1.08% total potassium) was 10 t hm^-2^. The oil residue fertilizer, derived from soybean cakes (organic matter 68%, total nitrogen 6.68%, total phosphorus 0.44%, total potassium 1.19%), was applied at a rate of 1.5 t hm^-2^. The chemical fertilizer application of urea 180 kg hm^-2^ (N, 46.4%), diammonium phosphate 150 kg hm^-2^ (N, 16%; P_2_O_5_, 44%) and potassium sulfate 225 kg hm^-2^ (K_2_O, 52%). In the treatment involving the application of CF, 65% of the chemical nitrogen fertilizer, along with all of the phosphate and potassium fertilizers, was blended as the basal fertilizer. Subsequently, during the heading stage, the remaining 35% of the chemical nitrogen fertilizer was applied as topdressing. The maize was irrigated in equal amounts by sprinkler irrigation. Irrigation once during the sowing period, with a dosage of 115.5 m^3^ hm^-2^. Irrigation twice during the seedling stage, with a dosage of 231 m^3^ hm^-2^. Irrigation three times during the jointing period, with a dosage of 1039.5 m^3^ hm^-2^. Irrigation twice during the heading period, with a dosage of 858 m^3^ hm^-2^. Irrigation once during the grouting period, with a dosage of 214.5 m^3^ hm^-2^.

**Table 1:** Physical and chemical properties of soft rock and sand.

Material	Sand (50-2000 μm) (%)	Silt (2-50 μm) (%)	Clay (<2 μm) (%)	pH	Capillary porosity (%)	Organic carbon (g kg^-1^)	CaO (%)	K_2_O (%)	SiO_2_ (%)
Soft rock	17.56	73.94	8.50	8.35	44.94	2.32	1.64	3.00	64.67
Sand	90.35	5.11	4.54	8.85	26.33	1.16	2.08	2.16	78.05

### 2.3 Soil sample collection

Soil samples were collected during the maize harvest on September 28, 2022. Initially, the aboveground maize was harvested, followed by the collection of rhizosphere soil samples. For the collection of rhizosphere soil, five maize plants were randomly selected from each experimental plot. The entire maize root system was meticulously excavated using a wooden shovel, taking care to remove sand, gravel, litter, and other debris. Soil located within 0-4 mm from the root surface was collected using the spading method. In each experimental plot, the rhizosphere soils from five maize plants were blended using the quartering technique to create a rhizosphere soil sample. From each test plot, three composite soil samples were collected, leading to a total of 36 soil samples (4 × 3 × 3). Each maize rhizosphere soil sample was subsequently transported to the laboratory and divided into three portions. One portion was stored in a 4°C refrigerator for soil organic carbon mineralization incubation and high-throughput sequencing, another portion for the analysis of soil physicochemical properties, and the remaining portion for assessing aggregate stability.

### 2.4 Organic carbon mineralization incubation

The mineralization of soil organic carbon was assessed using the lye absorption method, as described by Hu et al. (2023). The procedure entailed weighing 30.0 g of fresh samples for each treatment, positioning them at the base of a 1000 mL incubation bottle, adjusting the moisture content to around 60% using deionized water, and pre-incubating them in an incubator at 25°C for a period of 7 days. A 50 mL absorber, which contained a 10 mL solution of NaOH with a concentration of 0.1 mol L^-1^, was positioned at the base of the incubator and sealed. It was subsequently incubated in a dark environment at a temperature of 25°C. On the 1st, 3rd, 7th, 9th, 11th, 15th, 22nd, 25th, 32nd, 42nd, 50th, and 60th days of incubation, the alkaline liquid absorption cup was replaced, and water was added to the soil until a constant weight was reached. Next, 2 mL of a 1 mol L^-1^ BaCl_2_ solution was added to the absorption cup, followed by 2 drops of phenol indicator. The solution was titrated with a 0.1 mol L^-1^ HCl solution (calibrated with borax) until the red color disappeared.

### 2.5 Determination of soil physical and chemical properties

Soil organic carbon (SOC) was quantified using the K_2_Cr_2_O_7_-concentrated sulfuric acid and thermal oxidation method, total nitrogen (TN) content was assessed via Kjeldahl nitrometer analysis (Bao, 2000). Additionally, soil pH was measured with a pH meter, employing a soil-to-water ratio of 2.5:1 (Bao, 2000). Soil microbial biomass carbon (SMBC) was determined following the chloroform fumigation extraction method as described by Sam et al. (2002). The soil readily oxidizable carbon (ROC) content was determined using the potassium permanganate oxidation method, while the soil dissolved organic carbon (DOC) was quantified via the K_2_SO_4_ extraction method (Jeljli et al., 2022). Soil catalase activity was determined by potassium permanganate titration, phosphatase activity was determined by phenylene disodium phosphate colorimetry, and protease activity was determined by indenhydrin colorimetry (Qu et al., 2023; Tan et al., 2023).

### 2.6 Determination of soil aggregates

To assess the mechanical stability of soil aggregates, the dry sieving method was employed. In this process, 100 g of air-dried soil samples were extracted from each sample and deposited onto sieves with apertures of 2 mm, 1 mm, 0.5 mm, and 0.25 mm. Manual shaking of the sieve was carried out for a duration of 2-3 minutes. Subsequently, the soil aggregates on each layer of the sieve were gathered and individually weighed, following the protocol outlined by Cao et al. (2021). The water-stable aggregates were formed through the wet sieve method. A total of 20 g of soil aggregates for each particle size, previously obtained via the dry sieve method, were blended into a soil sample according to the dry sieve ratio. These amalgamated aggregates were then positioned on top of sieves with apertures measuring 2 mm, 1 mm, 0.5 mm, and 0.25 mm. The sieve was submerged in a water bucket, with the water level maintained at 2-3 cm above the top of the sieve, and allowed to stand undisturbed for a duration of 10 minutes. Following this, an aggregate analyzer was employed to agitate the sieve at a frequency of 25 times per minute for a duration of 3 minutes. The water-stable soil aggregates on each sieve layer were subsequently rinsed into aluminum containers, excess water was eliminated through settling, and the samples were subjected to drying at 105 °C before weighing (Zhang et al., 2021b).

### 2.7 High throughput sequencing

The Magabi Soil Genomic DNA Purification Kit was used to extract the total DNA of soil bacteria, and Thermo NanoDrop One was used to detect the purity and concentration of DNA. The PCR amplification was performed using universal primers 338F (5’-ACTCCTACG GGAGGCAGCA-3’) and 806R (5’-GACTACHVGGGTWTCTA AT-3’) for the V3-V4 variable regions of bacterial 16S rRNA genes. At ABI GeneAmp ^®^ On 9700, PCR amplification reaction was performed using the bacterial 16S rRNA gene reaction program, which was 95°C for 3 minutes, 94°C for 30 seconds, 30 cycles, and 72°C for 45 seconds. In various treatment groups, three samples were chosen for PCR amplification. Subsequently, the PCR products from each specific treatment group were combined and subjected to purification using a PCR product purification kit (Axigen Scientific, CA, USA). Then, Paired-end sequencing was performed using the Illumina MiSeq sequencing platform (Tao et al., 2020).

### 2.8 Analysis methods and data processing

Fitting equation of organic carbon mineralization parameters (Ribeiro et al., 2010),


(1) $$Ct=C_{o}(1-{\text{e}}^{-\text{k}t})$$

Where, *t* (d) is the incubation time, *C_t_* (mg kg^-1^) is the cumulative C mineralization after *t* time, C_0_ (mg kg^-1^) is the potential C mineralization in the soil, and k is the mineralization rate constant.

Soil aggregates with a size exceeding 0.25 mm play a pivotal role in soil fertility and serve as the fundamental building blocks of soil aggregate structure. In the context of this investigation, we evaluated the stability of aggregates larger than 0.25 mm, employing parameters such as the average weight diameter (MWD), geometric mean diameter (GMD), aggregate destruction rate (PAD), and aggregate stability rate (WASR) (Yang et al., 2022).


(2) $$\text{MWD}=\sum_{i=1}^{n}(\bar{d}iw\text{i})$$

(3) $$\text{GMD}=\text{exp}[\frac{\sum_{i=1}^{n}mi\ln\bar{d}i}{\sum_{i=1}^{n}mi}]$$

(4) $$\text{PAD}=\frac{{DR}_{0.25}-{WR}_{0.25}}{DR_{0.25}}\times 100\%$$

(5) $$\text{WASR}=\frac{WR_{0.25}}{DR_{0.25}}\times 100\%$$

Where, $\frac{—}{di}$ (mm) is the average diameter of soil aggregates in grade *i*, *w_i_* (%) is the percentage of soil aggregates in grade *i* to the total soil sample, *m_i_* (g) is the weight of soil aggregates in grade *i*, DR_0.25_ (%) is the proportion of soil mechanically stable aggregates >0.25 mm, and WR_0.25_ (%) is the proportion of soil water stable aggregates >0.25 mm.

The test data underwent initial processing through Microsoft Excel 2010 software. Subsequently, variance analysis and significance tests for differences in the test data were carried out using SPSS 23.0 statistical analysis software, employing the LSD method. Relevant graphs were constructed, and first-order dynamic equations were fitted using Origin 8.0 software. Additionally, a Pearson correlation analysis was performed. The software Circos-0.67-7 was employed for the generation of circular diagrams, known as Circos sample species relationship diagrams. These diagrams depict the interrelationship between samples and species. On the other hand, the Heatmap was utilized to represent data size within a two-dimensional matrix or table by utilizing color gradients. It offers insights into the composition and abundance of community species and can be analyzed using the R language (version 3.3.1) in conjunction with the vegan package. Alpha diversity analysis serves as a valuable tool for quantifying species richness, evenness, and overall diversity within a given community. Meanwhile, one-way ANOVA can be applied to assess disparities among groups. Redundancy analysis, conducted via the rda function within the R language’s vegan package, facilitates an intuitive visualization of the connection between sample distribution and environmental factors.

## 3 Results

### 3.1 Organic carbon mineralization process

**Figure 3:**
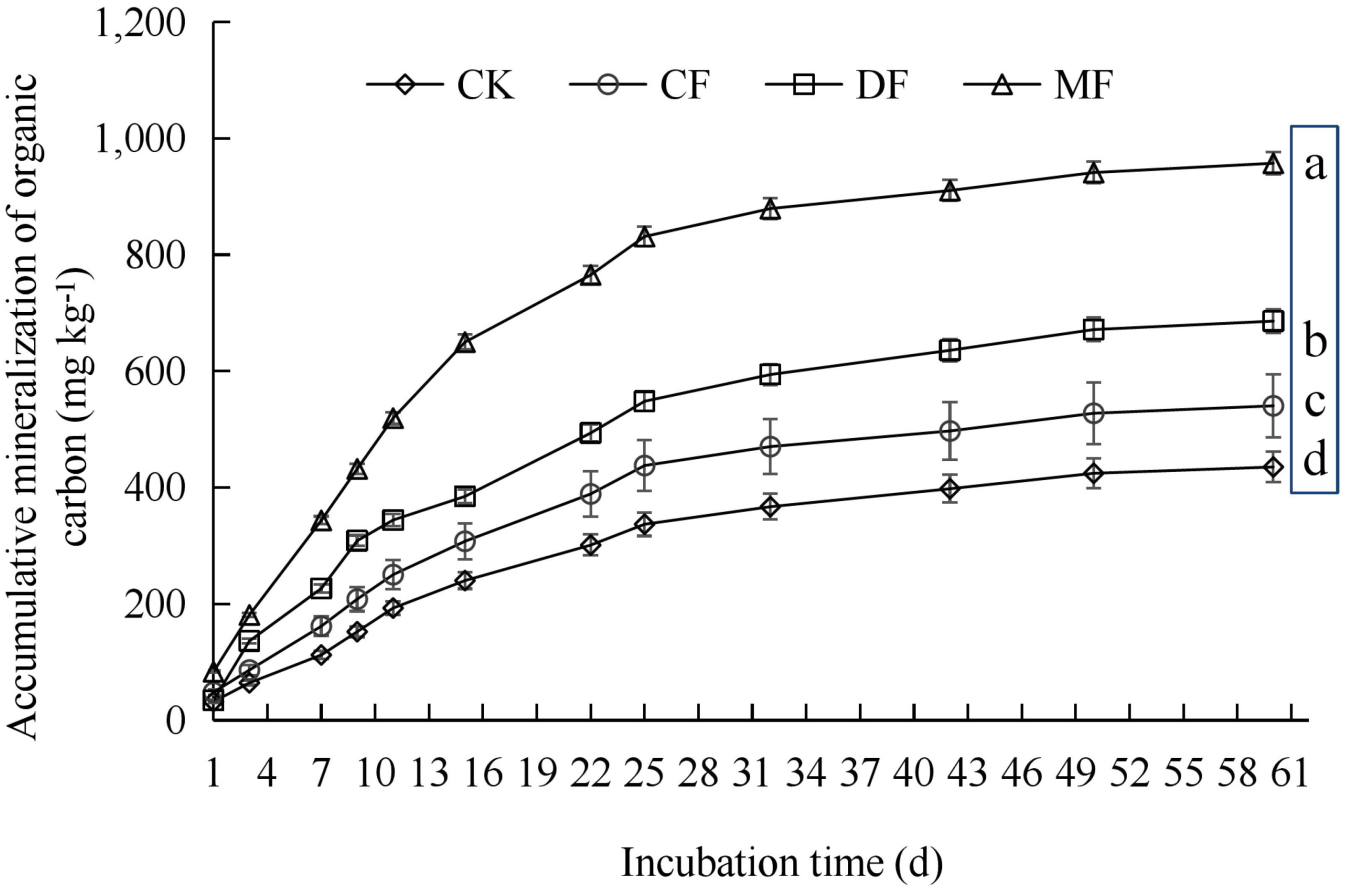
Cumulative mineralization of organic carbon in maize rhizosphere soil. CK means no fertilizer treatment, CF means fertilizer treatment, MF means cow manure treatment, DF means oil residue treatment. Lowercase letters indicate the significance test (P<0.05) of soil organic carbon cumulative mineralization at the end of 60 days of incubation.

**Figure 4:**
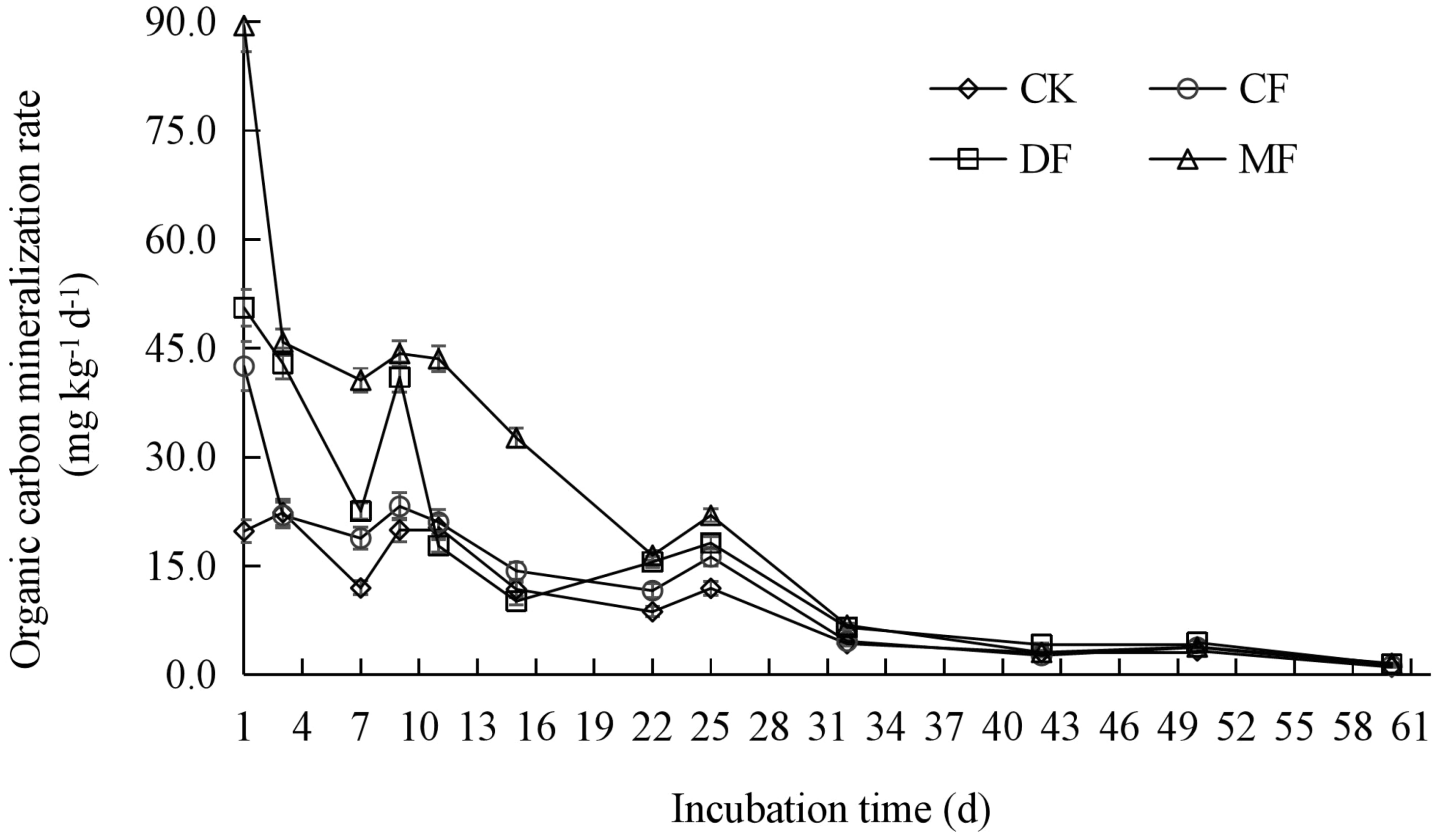
The mineralization rate of organic carbon in maize rhizosphere soil. CK means no fertilizer treatment, CF means fertilizer treatment, MF means cow manure treatment, DF means oil residue treatment.

Throughout various incubation periods, the cumulative mineralization of organic carbon in maize rhizosphere soil exhibited the following trend: MF > DF > CF > CK (**Figure 3**). At the conclusion of the incubation period, a notable disparity was observed in the cumulative mineralization of SOC among the various treatments. When compared to CK, the cumulative mineralization of SOC in the MF, DF, and CF treatments increased by 119.87%, 57.57%, and 24.15%, respectively (P<0.05). The cumulative mineralization of SOC remained consistent from the 9th to the 60th day, but no significant changes were observed in the initial 1st to 9th day period. The SOC mineralization can be generally categorized into two distinct stages. The initial stage spanned from 1st to 32nd and was characterized by a rapid mineralization process. On the 32nd day, there was a reduction in the mineralization rate by 78.22% to 92.34% when compared to the 1st (**Figure 4**). Subsequently, the second stage, which extended from 32nd to 60th, was marked by a slower rate of mineralization. By the 60th day, the mineralization rate had declined by 94.54% to 98.21% in comparison to the 1st.

### 3.2 Organic carbon mineralization parameters

Significant variations were observed in the gradient of SOC content and mineralization parameters among the different treatments. The fitting of kinetic equations demonstrated that all treatments exhibited highly significant differences (P<0.01), and the results of the fittings were considered valid (**Table 2**). The addition of exogenous fertilizers resulted in a substantial increase in SOC content. Specifically, the CF, DF, and MF treatments showed significant increases of 44.16%, 100.93%, and 145.79%, respectively, compared to the CK treatment (P<0.05). C_0_ exhibited a consistent trend with cumulative mineralization and SOC, and fertilization enhanced the potential mineralization capacity of organic carbon. Moreover, it was observed that the variation trend of k and T_1/2_ was inverse, whereby higher k values corresponded to smaller T_1/2_ values. The results revealed a substantial increase in the k values for the DF and MF treatments, with increments of 23.52% and 45.97%, respectively, compared to the CK treatment. Additionally, the T_1/2_ values exhibited a notable decrease of 19.06% and 31.52%. As for C_0_/SOC, representing the mineralization intensity of unit organic carbon, it was found to be the lowest in the DF treatment, significantly lower than in the other treatments by 14.96% to 25.02%.

**Table 2:** Soil organic carbon content and mineralization parameters.

Treatments	SOC (g kg-1)	C0 (mg kg-1)	k (d-1)	T1/2 (d)	C_0_/SOC (%)	R2
CK	4.28 ± 0.11 d	467.13 ± 9.71 d	0.0472 ± 0.0022 b	14.69 ± 2.53 a	10.91 ± 1.06 a	0.9959**
CF	6.17 ± 0.54 c	567.31 ± 10.22 c	0.0531 ± 0.0023 ab	13.05 ± 1.96 a	9.19 ± 1.93 b	0.9960**
DF	8.60 ± 1.02 b	703.42 ± 12.56 b	0.0583 ± 0.0026 a	11.89 ± 2.05 b	8.18 ± 0.74 c	0.9952**
MF	10.52 ± 0.96 a	978.31 ± 12.59 a	0.0689 ± 0.0024 a	10.06 ± 0.75 b	9.30 ± 1.26 b	0.9966**

SOC is the content of soil organic carboin, C_0_ is the potential mineralizable C, k is the SOC mineralization rate constant, T_1/2_ is the half-cycle period. Numbers with various characters in one column represent a substantial difference of 0.05 between treatments, while **indicates a extremely significant at 0.01 level. Lowercase letters indicate significant differences between treatments (P < 0.05).

### 3.3 Soil nutrient characteristics and enzyme activity

The application of organic material treatments led to a comprehensive increase in soil nutrient content and enzyme activity, as illustrated in **Table 3**. Notably, the TN content and catalase activity followed a trend of DF>MF>CF>CK, with significant differences observed among the various treatments (P<0.05). In particular, the MF treatment notably increased the SMBC content, with an increase of 83.33%, 56.44%, and 30.93% in comparison to the CK, CF, and DF treatments, respectively. Regarding the ROC content, there was no significant difference between the DF and MF treatments, both of which exhibited significantly higher levels compared to the CK and CF treatments. Similarly, there was no substantial variation in DOC content between the DF and MF treatments, although the MF treatment yielded significantly superior results compared to the CK and CF treatments. Phosphatase activity and protease activity exhibited consistent patterns among the CF, DF, and MF treatments, with both MF and DF treatments demonstrating significantly higher activity levels compared to the CK treatment. It’s worth noting that the soil pH remained within the range of weak alkalinity, and there were no significant differences in pH among the different treatments.

**Table 3:** Soil physical and chemical properties.

Treatments	TN (g kg-1)	SMBC (mg kg-1)	ROC (g kg-1)	DOC (mg kg-1)	Catalase (mg g-1)	Phosphatase (mg g-1)	Protease (mg g-1)	pH
CK	0.40 ± 0.11 d	60.22 ± 4.51 c	0.62 ± 0.05 b	6.20 ± 1.05 c	16.74 ± 2.20 d	0.30 ± 0.01 b	0.81 ± 0.17 b	8.99 ± 1.28 a
CF	0.58 ± 0.03 c	70.57 ± 6.90 bc	0.68 ± 0.03 b	7.55 ± 1.47 b	20.89 ± 3.91 c	0.38 ± 0.02 ab	1.05 ± 0.04 ab	9.05 ± 0.60 a
DF	0.92 ± 0.03 a	84.32 ± 2.74 b	1.02 ± 0.11 a	8.72 ± 1.21 ab	29.76 ± 3.84 a	0.42 ± 0.12 a	1.56 ± 0.20 a	8.75 ± 1.62 a
MF	0.75 ± 0.15 b	110.40 ± 8.06 a	0.92 ± 0.14 a	10.02 ± 1.44 a	24.13 ± 2.40 b	0.50 ± 0.02 a	1.44 ± 0.15 a	8.90 ± 0.91 a

TN is the content of total nitrogen, SMBC is the soil microbial biomass carbon, ROC is the soil easily oxidized organic carbon, DOC is the soil dissolved organic carbon, numbers with various characters in one column represent a substantial difference of 0.05 between treatments. Lowercase letters indicate significant differences between treatments (P < 0.05).

### 3.4 Soil aggregate diameter

The MWD and GMD of mechanically stable aggregates were consistently higher under the various treatments in comparison to water-stable aggregates (**Table 4**). However, when using the dry screening method, there was no significant difference in MWD among the treatments (P>0.05). Notably, in comparison to the CK treatment, the GMD increase was most prominent under the DF treatment, showing a substantial increase of 50.84% (P<0.05). When employing the wet screening method, there were no significant differences in GMD among the different treatments. However, compared to the CK treatment, the CF, DF, and MF treatments displayed significant increases of 26.22%, 24.45%, and 22.62%, respectively. Notably, there were no significant distinctions observed between the CF, DF, and MF treatments. It was observed that the GMD index exhibited greater sensitivity to changes under the dry screening method, while the MWD index was more responsive to changes under the wet screening method. Specifically, the DF treatment was found to be effective in increasing the diameter of soil aggregates.

**Table 4:** Soil physical and chemical properties.

Treatments	Mechanical stability aggregates		Water stable aggregates	
	MWD (mm)	GMD (mm)	MWD (mm)	GMD (mm)
CK	0.2660 ± 0.02 a	0.2486 ± 0.01 bc	0.1861 ± 0.01 b	0.1588 ± 0.02 a
CF	0.2894 ± 0.03 a	0.2921 ± 0.04 ab	0.2349 ± 0.06 a	0.1763 ± 0.07 a
DF	0.2372 ± 0.05 ab	0.3750 ± 0.10 a	0.2316 ± 0.03 a	0.1740 ± 0.05 a
MF	0.2713 ± 0.01 a	0.2798 ± 0.06 b	0.2282 ± 0.04 a	0.1780 ± 0.02 a

MWD (mm) represents the average weight diameter, GMD (mm) represents the geometric mean diameter. The data in the table is the mean ± standard deviation. Different lowercase letters in the same column indicate significant differences between different treatments (P<0.05).

### 3.5 Soil aggregate stability

**Figure 5:**
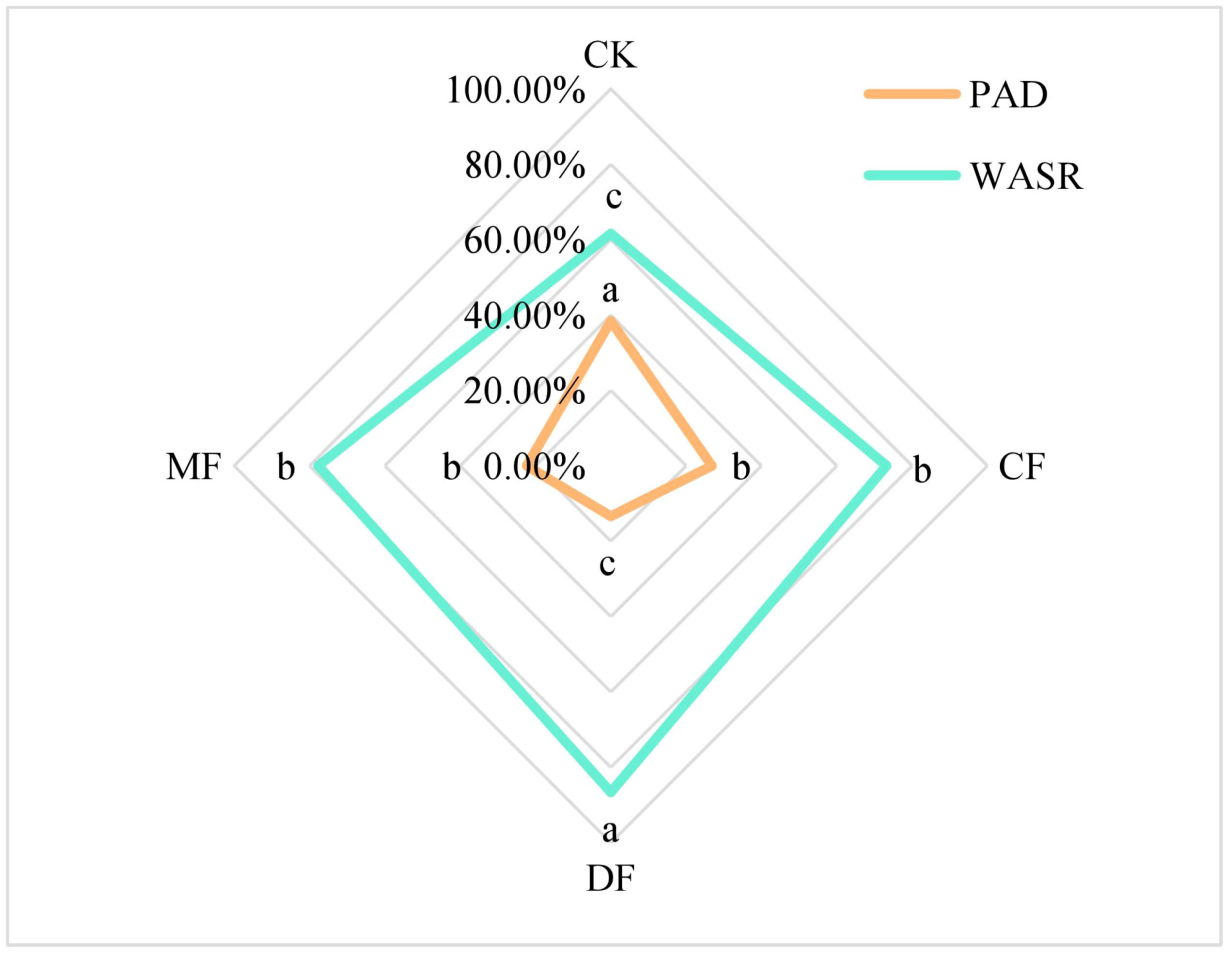
Stability parameters of soil aggregates. PAD represents the aggregate destruction rate, and WASR represents the aggregate stability rate. CK means no fertilizer treatment, CF means fertilizer treatment, MF means cow manure treatment, DF means oil residue treatment. Lowercase letters indicate significant differences at the 5% level between different treatments.

PAD and WASR are key indicators used to evaluate the rate of soil aggregate destruction and stability, respectively. These indicators offer valuable insights into the overall stability of soil aggregates. A higher PAD value signifies a greater level of instability in soil aggregates, while a larger WASR value indicates enhanced stability. The findings of this study revealed that the PAD value in the CK treatment was the highest, followed by the CF and MF treatments, with the DF treatment exhibiting the lowest PAD value. Specifically, when compared to the CK treatment, the DF treatment significantly reduced PAD by 65.13% (P<0.05). In comparison to the CF and MF treatments, the reduction in PAD was notably higher in the DF treatment, with a percent decrease of 51.85% and 40.91%, respectively (P<0.05). Conversely, the pattern observed for WASR exhibited an opposite trend. The WASR of the DF treatment was the highest, showing an increase ranging from 11.49% to 40.60% in comparison to the other treatments (P<0.05). This indicates that the aggregates stability under the DF treatment was significantly stronger (**Figure 5**).

### 3.6 The relationship between soil physicochemical properties and organic carbon mineralization

The analysis of Pearson correlations among soil physicochemical properties, enzyme activity indicators, and organic carbon mineralization parameters unveiled several key relationships. SOC, SMBC, and DOC showed a notable positive impact on organic carbon mineralization (P<0.05). However, these factors displayed a significant negative correlation with the turnover time of organic carbon. Among enzyme activity indicators, only phosphatase activity exhibited a significant correlation with organic carbon mineralization parameters. In the context of aggregates, the soil mineralization rate per unit of organic carbon (C_0_/SOC) demonstrated a significant positive correlation with PAD and a significant negative correlation with WASR (**Table 5**).

**Table 5:** The Pearson correlation between soil physicochemical properties and organic carbon mineralization.

Index	C_t_ (mg kg^-1^)	C_0_ (mg kg^-1^)	k (d^-1^)	T_1/2_ (d)	C_0_/SOC (%)
SOC (g kg^-1^)	0.9731*	0.9685*	0.9804*	-0.9918*	-0.6729
SMBC (mg kg^-1^)	0.9999*	0.9998*	0.9975*	-0.9832*	-0.4917
DOC (mg kg^-1^)	0.9751*	0.9711*	0.9859*	-0.9980*	-0.6701
Phosphatase (mg g^-1^)	0.9746*	0.9719*	0.9884*	-0.9985*	-0.6426
PAD (%)	-0.6119	-0.5965	-0.6447	0.7240	0.9734*
WASR (%)	0.6119	0.5965	0.6447	-0.7240	-0.9734*

SOC is the content of soil organic carboin, SMBC is the soil microbial biomass carbon, DOC is the soil dissolved organic carbon, PAD represents the aggregate destruction rate, and WASR represents the aggregate stability rate, C_t_ is the accumulated C mineralization at the end of incubation, C_0_ is the potential C mineralization, k is the SOC mineralization rate constant, T_1/2_ is the half-cycle period. *indicates a significant correlation at the 5% level.

### 3.7 Composition of bacterial community

**Figure 6:**
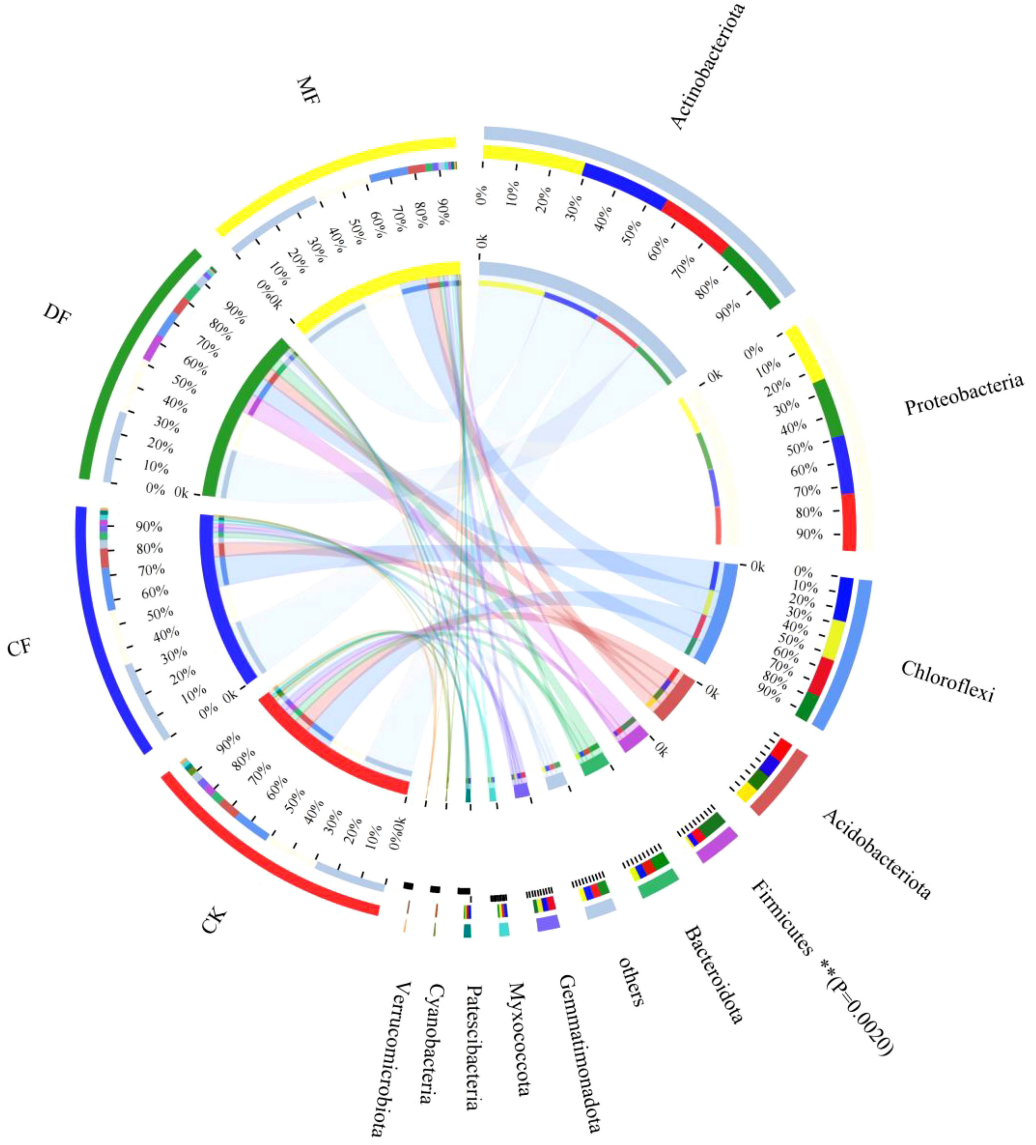
Composition of soil bacterial communities at the Phulum level. CK means no fertilizer treatment, CF means fertilizer treatment, MF means cow manure treatment, DF means oil residue treatment. **indicates that the bacterial abundance reached a significant level of 1% between treatments.

At the phyla classification level, the species with bacterial abundance greater than 1% were mainly distributed in *Actinobacteriota*, *Proteobacteria*, *Chloroflexi*, *Acidobacteriota*, *Firmicutes*, *Bacteroidota*, *Gemmatimonadota*, *Myxococcota*, *Patescibacteria*, *Verrucomicrobiota* and *Cyanobacteri*a. Among them, *Actinobacteriota*, *Proteobacteria*, *Chloroflexi*, *Acidobacteriota* and *Firmicutes* accounted for 80.54% to 88.13% of the total number of bacteria, making them the dominant community in the rhizosphere soil (**Figure 6**). The abundance of *Firmicutes* exhibited a significant difference among treatments (P<0.01). Specifically, in the DF treatment, the abundance of *Firmicutes* increased by 194.42% in comparison to the CK treatment.

**Figure 7:**
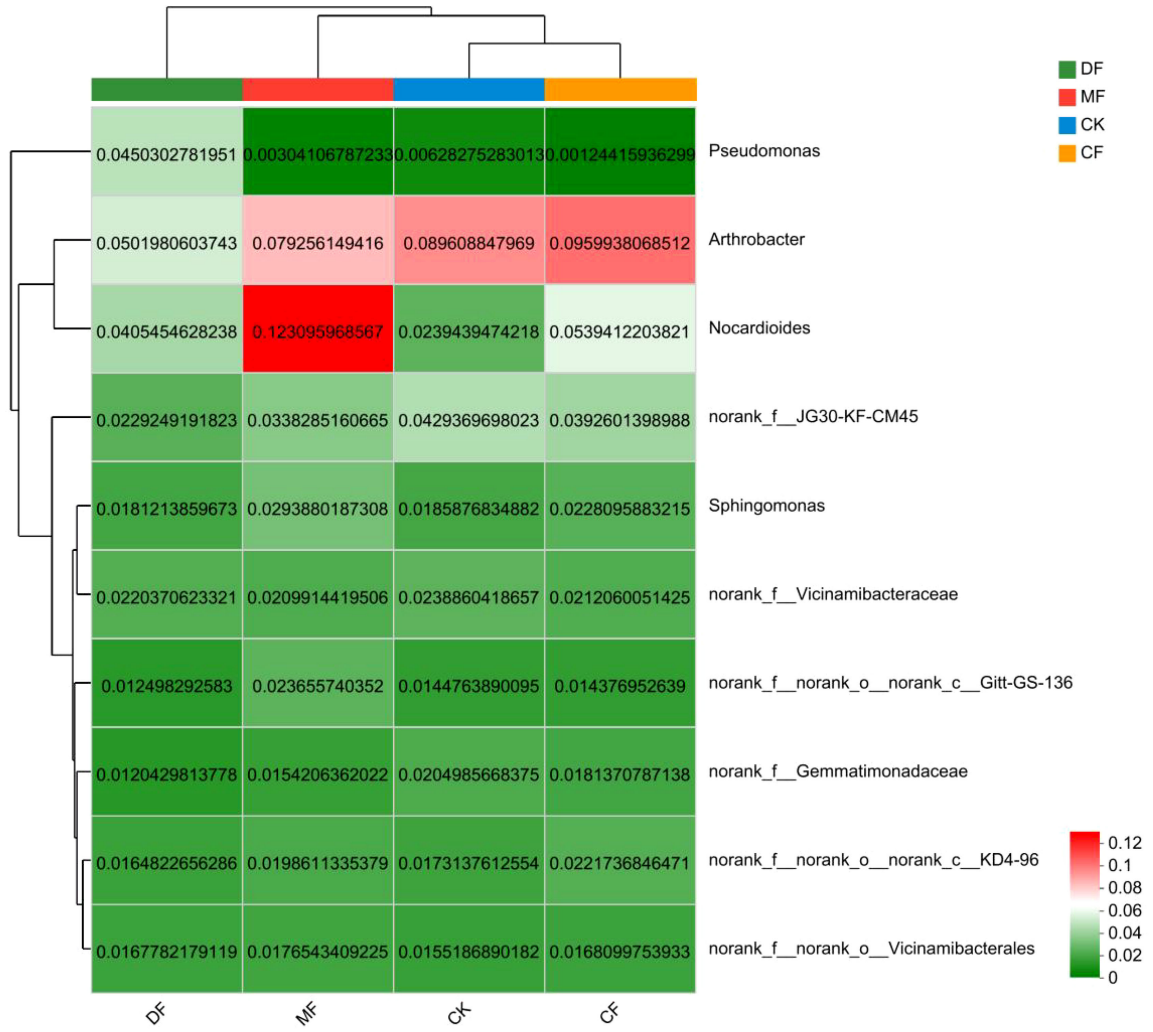
Community heatmap analysis on Genus level. CK means no fertilizer treatment, CF means fertilizer treatment, MF means cow manure treatment, DF means oil residue treatment. The horizontal axis represents the sample name, and the vertical axis represents the species name. The abundance of different species is displayed through a color gradient of color blocks.

At the genus classification level, the bacterial species composition displayed increased diversity, with a higher count of unnamed species. Intriguingly, a substantial proportion, sixty percent, of the top 10 species with the highest abundance remained unclassified. *Arthrobacter* and *Nocardioides* genera were classified in the *Actinobacteriota* phylum, while *Sphingomonas* and *Pseudomonas* genera were classified in the *Pseudomonadota* phylum. The species clustering in **Figure 7** also categorizes *Arthroactors* and *Nocardioides* into one category. In CK and CF treatments, species richness showed a trend of *Arthroactor*>*Nocardioides*>*Sphingomonas*>*Pseudomonas*. In DF treatment, the species richness was displayed as *Arthroactor*>*Pseudomonas*>*Nocardioides*>*Sphingomonas*. In MF treatment, the species richness was *Nocardioides*>*Arthroactor*>*Sphingomonas*>*Pseudomonas*. The species richness composition has been noted to vary under different treatments. Cluster analysis has delineated three distinct categories for fertilization treatments, with CK and CF forming one cluster, and DF and MF grouped separately. This observation implies that fertilization exerts a substantial influence on the species composition of bacteria.

### 3.8 Bacterial Alpha diversity in rhizosphere soil

**Figure 8:**
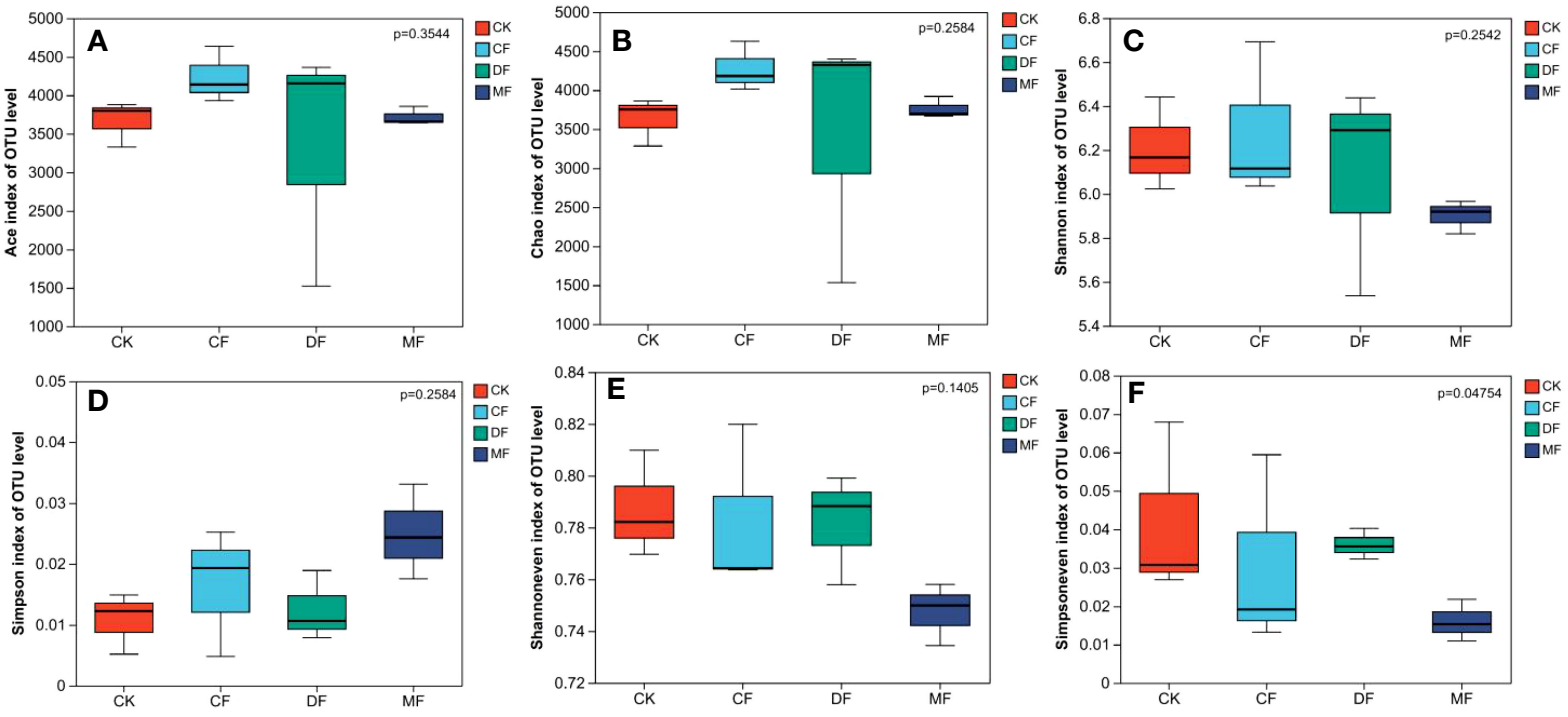
The bacterial Alpha diversity analysis. **(A)** is the ACE index, **(B)** is the Chao index, **(C)** is the Shannon index, **(D)** is the Simpson index, **(E)** is the Shannoneven index, **(F)** is the Simpsoneven index. CK means no fertilizer treatment, CF means fertilizer treatment, MF means cow manure treatment, DF means oil residue treatment.

The bacterial richness of rhizosphere soil increased following CF and DF treatments, while the MF treatment resulted in decreased richness. When compared to the CK treatment, the Ace index and Chao index exhibited increments ranging from 8.97% to 9.43% and 11.47% to 15.23%, respectively. Although the species diversity index displayed no significant differences among treatments, the DF treatment still showed an increasing trend, with the Shannon index increasing by 1.94% compared to the CK treatment. Furthermore, both CF and MF treatments reduced the uniformity index of soil bacteria, while the DF treatment enhanced it. The Shanoneven index and Simpsoneven index of the DF treatment increased by 0.78% and 15.64%, respectively, compared to the CK treatment. Notably, there was a significant inter-group difference in the Simpsoneven index (P<0.05) (**Figure 8**).

### 3.9 Redundancy analysis of soil bacteria and organic carbon mineralization

**Figure 9:**
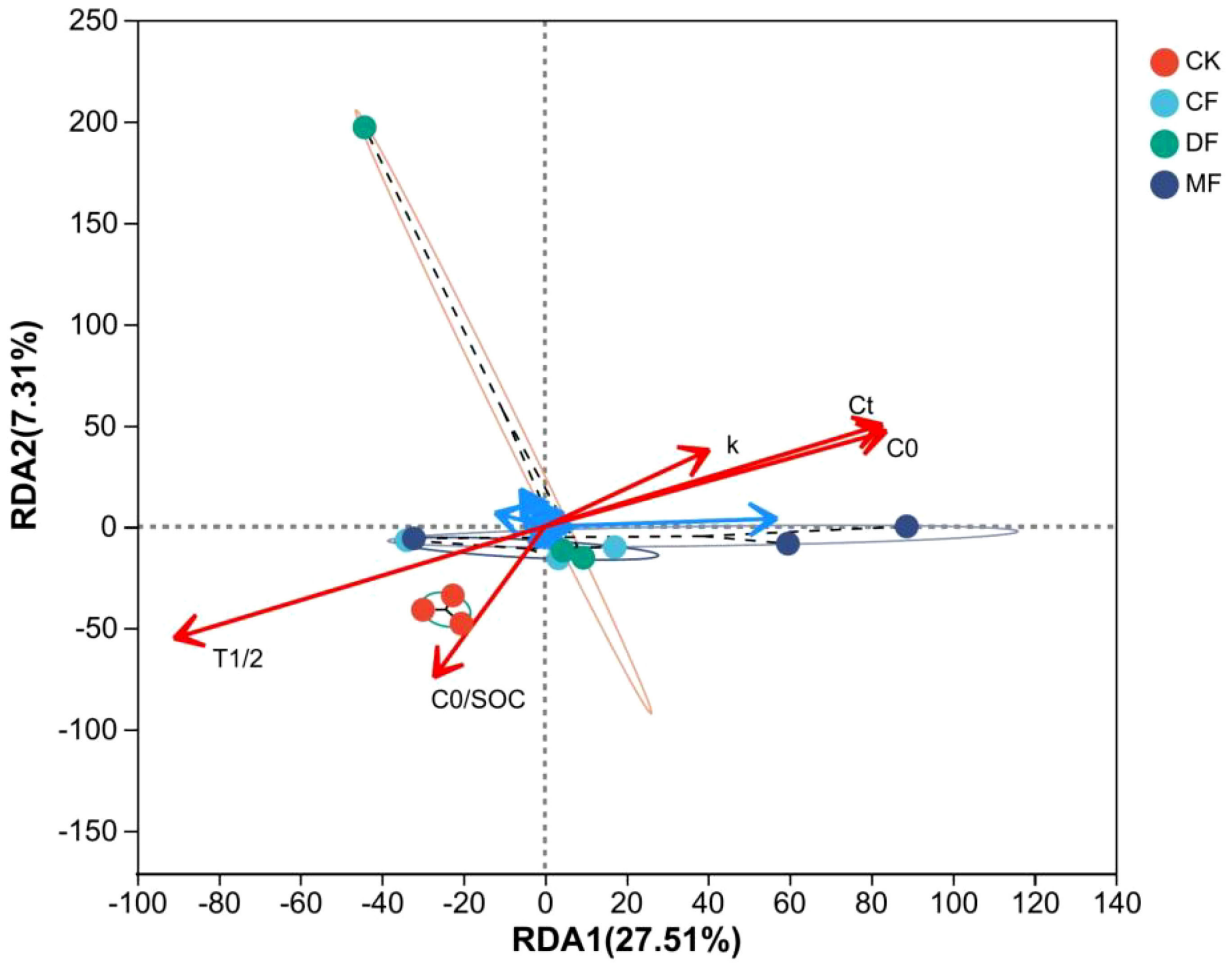
The redundancy analysis of soil bacteria and organic carbon mineralization. CK means no fertilizer treatment, CF means fertilizer treatment, MF means cow manure treatment, DF means oil residue treatment. SOC represents the soil organic carbon content, C_t_ stands for the accumulated C mineralization at the conclusion of incubation, while C_0_ represents the potential C mineralization. The parameter k signifies the SOC mineralization rate constant, and T_1/2_ represents the half-cycle period.

**Figure 10:**
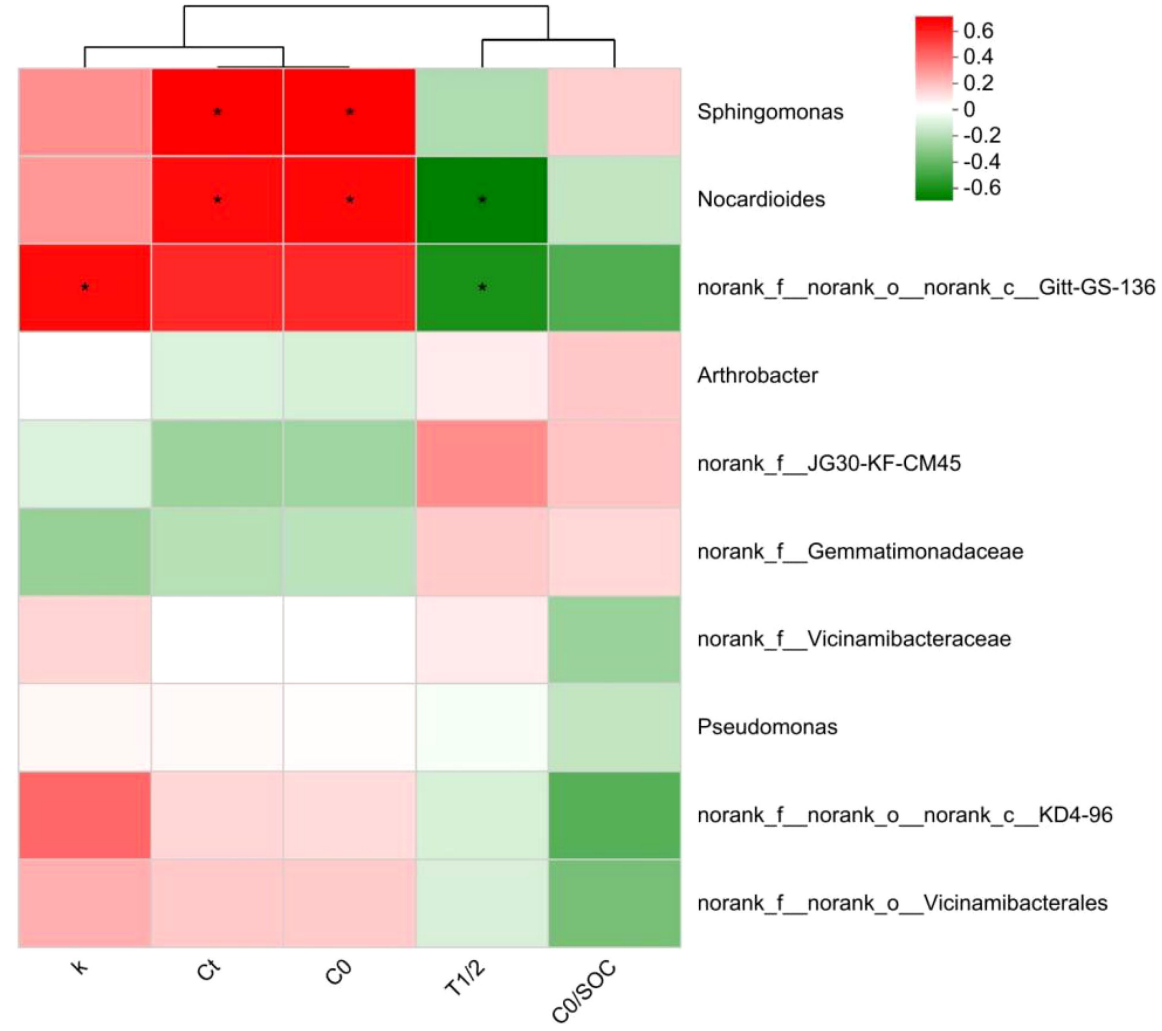
The correlation heatmap between bacterial community and organic carbon mineralization parameters. SOC represents the soil organic carbon content, C_t_ stands for the accumulated C mineralization at the conclusion of incubation, while C_0_ represents the potential C mineralization. The parameter k signifies the SOC mineralization rate constant, and T_1/2_ represents the half-cycle period. *indicates a significant correlation at the 5% level.

The study unveiled a significant correlation between the soil bacterial community and parameters associated with organic carbon mineralization. The results indicated that the bacterial community exerted an influence on organic carbon mineralization parameters, with T_1/2_ being the most influential, followed by C_0_, C_t_, k, and C_0_/SOC. Notably, T_1/2_ exhibited a significant difference. The turnover time of organic carbon in soil was found to have a strong correlation with the bacterial community, as shown in **Figure 9**. *Nocardioides* (belonging to *Actinobacteriota*) and *norank_f:norank_o:norank_c:Gitt-GS-136* showed a significant negative correlation with the organic carbon half turnover period (T_1/2_). The *norank_f:norank_o:norank_c:Gitt-GS-136* showed a significant positive correlation with k. The *Sphingomonas* (belonging to phylum *Pseudomonadota*) and *Nocardioides* (belonging to phylum *Actinobacteriota*) significantly promoted the amount of organic carbon mineralization (C_t_ and C_0_) and showed a significant correlation with each other (**Figure 10**).

## 4 Discussion

### 4.1 The relationship between maize rhizosphere soil nutrients and organic carbon mineralization

The preceding study observed that pure sand exhibited the lowest organic carbon mineralization rate in comparison to compounded soil. When the ratio of soft rock to sand ranged from 1:5 to 1:2, there was a significant increase in the retention time of SOC (Guo et al., 2019b). The application of organic fertilizer had a pronounced influence on both soil organic carbon mineralization and nutrient characteristics (Qi et al., 2016; Guo, 2019; Xu et al., 2021). This study’s results unveiled noticeable distinctions in the cumulative mineralization of maize rhizosphere SOC under various fertilizer treatments. The MF treatment displayed the highest cumulative mineralization, followed by the DF and CF treatment, with the lowest cumulative mineralization observed in the CK treatment. Furthermore, a notable increase in maize rhizosphere SOC content was observed. Similar studies have reported that organic fertilizer application significantly enhances SOC mineralization rates and nutrient content (Cookson et al., 2005; Manna et al., 2007), which aligns with our findings. The increase in soil nutrient content can be attributed to the input of organic matter and nutrients from organic fertilizers, providing energy and nutrients to the soil microbial community (Ribeiro et al., 2010). Maize, as a C4 plant, exhibits unique photosynthetic characteristics. The maize roots release a range of organic compounds, including root exudates and mucilage, into the rhizosphere. These organic compounds serve as an energy source for soil microorganisms and, in turn, influence the mineralization of organic carbon (Abdullah and Shagufta, 2016). Consequently, the DF and MF treatments exhibited higher SOC and TN content (**Table 3**). The promotion of SOC, SMBC, and DOC content significantly enhanced the organic carbon mineralization process (**Table 5**). Previous studies by Yan et al. (2007) also confirmed that organic fertilizers effectively promote the accumulation of light fraction organic carbon and microbial biomass carbon, supporting our study’s findings. This affirms that the application of organic fertilizers can enhance active carbon levels in the maize rhizosphere soil, thereby further stimulating organic carbon mineralization. In this investigation, the C_0_/SOC ratio in the DF treatment was determined to be the lowest, signifying that DF increased nutrient content and promoted mineralization while demonstrating the lowest organic carbon loss per unit. This implies a more favorable indirect carbon sequestration effect. Oil residue fertilizers are renowned for their rich organic matter content. When incorporated into the soil, they supply a substantial amount of carbon sources and nutrients, which enhance soil microbial activity and foster the degradation and transformation processes of organic matter (Comte et al., 2013; Guo et al., 2019a). It is worth noting that specific components in oil residue fertilizers, such as fatty acids and sugars, may have inhibitory effects on soil microbial activity, potentially decelerating the decomposition of organic matter.

### 4.2 The effect of soil agglomeration structure on organic carbon mineralization

This study focuses on composite soil, comprising a mixture of soft rock and sand, characterized as a non-uniform medium soil. Our research represents the first comprehensive exploration of organic carbon mineralization in this distinctive compound soil in the context of organic fertilization. The findings of this study provide novel insights and directions for investigating similar soil types. Our study revealed a positive correlation between C_0_/SOC and PAD while showing a negative correlation with WASR. These outcomes suggest that enhancing aggregate stability in maize rhizosphere soil can potentially reduce the mineralization rate of unit organic carbon and facilitate carbon sequestration. A study by Chen et al. (2022) also found that the application of organic fertilizers significantly improved the structural stability of soil aggregates in maize rhizosphere soil, and this enhancement further supported the sequestration of soil organic carbon. These results align with our observations, underlining the beneficial impact of organic fertilizers on the mechanisms involved in SOC mineralization in the context of maize cultivation. Furthermore, in our investigation, the application of the DF treatment resulted in enhanced aggregate stability and a larger GMD of mechanically stable aggregates within the maize rhizosphere. These aggregate characteristics have the potential to enhance soil aeration and permeability, increase soil porosity, and facilitate the entry of water and oxygen. Additionally, the organic matter present in organic fertilizers can act as a binding agent for soil particles in maize rhizosphere soil, thereby enhancing the stability of soil aggregates (Jiang et al., 2021). This, in turn, reduces the loss of soil organic carbon due to water and wind erosion and promotes the mineralization process of soil organic carbon, ultimately benefiting maize growth and overall soil health (Chen et al., 2022; Yang et al., 2022).

### 4.3 Response of maize rhizosphere SOC mineralization to microorganisms

The relationship between soil bacterial communities and parameters of organic carbon mineralization is of great significance, particularly within the context of maize rhizosphere soil. The maize is a vital global cereal crop, and understanding its interaction with soil microorganisms and organic carbon mineralization is essential for sustainable agriculture. In this regard, organic fertilizers play a pivotal role by enhancing soil microbial activity within the maize rhizosphere. This heightened microbial activity, as evidenced by prior research, leads to the acceleration of the decomposition and mineralization of organic matter (Liu et al., 2021). The CF, DF, and MF treatments produced significant alterations in the bacterial community structure, with the CF treatment exhibiting a community structure similar to that of the CK treatment (**Figure 6**). Furthermore, the application of organic fertilizers led to an increase in the relative abundance of specific bacteria, such as the genus *Nocardioides* (belonging to the Phylum *Actinobacteriota*). In particular, the abundance of *Nocardioides* in the DF and MF treatments increased by 69.45% and 415.06%, respectively, compared to the CK treatment. These organic fertilizer treatments notably enhanced the evenness index of soil bacteria, although there was no discernible difference in the diversity index. Interestingly, a European study by Geisseler and Scow (2014) reported significant alterations in soil bacterial communities and an increase in bacterial diversity upon the application of organic fertilizers, which appears to contradict the findings of this study. This suggests a shift in the community structure toward certain bacterial groups without necessarily increasing overall diversity. While these findings are consistent with the notion that organic fertilizers promote the growth of specific microorganisms, they also highlight the unique role of *Nocardioides* in the maize rhizosphere. *Nocardioides*, as indicated by this study, exerts a noteworthy stimulating effect on the mineralization of organic carbon. This supports the idea that certain microbial taxa within the maize rhizosphere may play a crucial role in organic carbon mineralization processes.

In summary, the application of organic fertilizers can improve the comprehensive properties of soil and promote the mineralization process of organic carbon. However, the specific effects are influenced by soil environmental conditions, so it is necessary to develop appropriate fertilization strategies based on different soil types and management practices to maximize the benefits of organic fertilizers.

## 5 Conclusion

Various fertilizer treatments have been assessed for their impact on a range of soil attributes, including the SOC mineralization process, nutrient characteristics, enzyme activity, soil aggregate dimensions, stability, bacterial community composition, and bacterial diversity in the maize rhizosphere. The application of these fertilizers has been observed to enhance the mineralization of soil organic carbon while concurrently reducing the turnover time of carbon. Concurrently, the utilization of oil residue fertilizer results in a decreased rate of organic carbon loss, contributing indirectly to carbon sequestration. Furthermore, the application of oil residue fertilizer significantly amplifies the concentration of active organic carbon and enzyme activity in the soil, thereby enhancing soil fertility. In addition, the use of oil residue fertilizer leads to a marked increase in soil aggregate size, thereby bolstering soil structural stability. The composition, abundance, and even distribution of bacterial communities exhibit variations across different treatment regimes. Particularly noteworthy is the observed increase in *Nocardioides* abundance, which can enhance the organic carbon mineralization process. These findings bear substantial significance in comprehending the impact of organic amendments on soil functions, offering valuable theoretical and practical insights for soil management and the advancement of sustainable agricultural practices.

## Data availability statement

The datasets presented in this study can be found in online repositories. The names of the repository/repositories and accession number(s) can be found below: GenBank, PRJNA984686.

## Author contributions

ZG: Conceptualization, Formal analysis, Funding acquisition, Software, Validation, Writing – original draft. JH: Conceptualization, Formal analysis, Funding acquisition, Software, Validation, Writing – original draft. YZ: Conceptualization, Project administration, Supervision, Writing – review & editing. HW: Conceptualization, Data curation, Validation, Writing – review & editing.
